# Implementation of Disaster Prevention Education and Maternal and Child Health Handbook Guidance for Pregnant Women in Japanese Medical Institutions: A Pilot Study

**DOI:** 10.3390/nursrep16020071

**Published:** 2026-02-18

**Authors:** Haruka Iida, Hisao Nakai, Nobuki Shimaoka, Masayo Takada

**Affiliations:** 1 Department of Nursing, Faculty of Nursing, University of Kochi, 2751-1 Ike, Kochi 781-8515, Japan; iida_haruka@cc.u-kochi.ac.jp (H.I.); shimaoka@cc.u-kochi.ac.jp (N.S.); 2 Department of Nursing, Faculty of Health and Social Services, Kanagawa University of Human Services, 1-10-1 Heisei-cho, Kanagawa, Yokosuka 238-8522, Japan; gmmtakada@gmail.com

**Keywords:** disaster prevention education, Maternal and Child Health Handbook, midwife, pregnant women

## Abstract

**Background:** In disaster-prone countries like Japan, disaster prevention education (DPE) is considered essential for vulnerable populations, including pregnant women. This pilot study aimed to clarify the status of DPE and the use of the Maternal and Child Health Handbook (MCHH) in medical institutions during disasters. **Methods:** This cross-sectional pilot study was conducted in 2020. An anonymous self-administered questionnaire was distributed to administrators at 101 medical facilities across three Japanese prefectures. Descriptive statistics and Fisher’s exact test were used to analyze the implementation of DPE and associated factors, focusing on MCHH guidance (medical history, identification, and carrying the handbook). **Results:** Of the 28 facilities with valid responses, 9 (32.1%) implemented DPE. There was a tendency for guidance on MCHH usage during disasters to be provided in facilities that were General or Regional Perinatal Medical Centers (*n* = 5, 83.3%; *p* = 0.007). Despite limited sample sizes in some categories, the results suggested that specific institutional characteristics influence the provision of disaster preparedness information. **Conclusions:** While perinatal medical centers are multifaceted and busy, they play an essential role in emphasizing the importance of the MCHH during disasters. Facilities already providing DPE should integrate broader preparedness measures alongside specific MCHH guidance to improve maternal and child safety.

## 1. Introduction

Global climate change is increasing the frequency and severity of natural disasters, creating substantial public health challenges worldwide [1,2,3]. According to the Emergency Events Database (EM-DAT), more than 300 to 400 natural disasters are recorded annually worldwide, affecting millions of people and causing extensive economic damage [4]. This global trend highlights the urgent need for effective disaster preparedness strategies for vulnerable populations, including pregnant women, who face unique health risks during such disasters.

Since 2011, major earthquakes have occurred every few years in Japan; these include the Great East Japan Earthquake in 2011, the Kumamoto Earthquake in 2016, the Hokkaido Eastern Iburi Earthquake in 2018, and the Noto Peninsula Earthquake in 2024 [5]. Furthermore, human casualties owing to extreme torrential rain frequently occur [6], such as the disaster in western Japan in July 2018 [7], the widespread disaster that occurred from the Kyushu to the Tohoku regions in July 2020 [8], and the disasters associated with the landfall of typhoons such as Typhoon Hagibis (No. 19) in 2019 [9]. Consequently, the need for evacuation has been increasing.

Disasters have a substantial effect on the health and well-being of the population, including pregnant women. Some women who were pregnant at the time of or immediately following the Great East Japan Earthquake and the 2011 Fukushima nuclear accident (which occurred following a major earthquake and tsunami) experienced increased pregnancy complications such as hypertensive disorders, respiratory diseases, and mental disorders [10]. During the hospital triage following the February 2023 Turkish earthquake, severely injured patients received priority. Consequently, pregnant women were given lower priority, resulting in insufficient time allocated to their care and inadequate psychological support [11]. Furthermore, reports from Indonesia indicate that pregnant women are susceptible to issues such as nutritional deficiencies and inequities in disaster situations [12]. The psychological and physical effects of exposure to hazards such as natural disasters, including stress and inadequate nutrition, negatively affect birth outcomes, and can lead to preterm birth and low infant birth weight [13,14]. Notably, exposure to earthquakes during pregnancy is associated with substantial reductions in average birth weight, birth length, and head circumference, as well as increased rates of preterm birth, miscarriage, and stillbirth [15]. Moreover, evidence suggests that mothers affected by earthquakes may experience worsened mental health as a result, which can increase negative perinatal outcomes such as postpartum depression and post-traumatic stress [16,17]. Exposure to hurricanes and floods can also have a substantial negative effect on pregnant women. For instance, following Hurricane Michael, which struck the southeastern United States in 2018, delays in initiating prenatal care and reduced rates of antenatal visits among pregnant women were reported [18]. Furthermore, exposure to hurricanes during pregnancy negatively affects pregnancy outcomes [19,20]. Considering the risk of these adverse effects owing to disasters and related environmental conditions, it is recommended that pregnant women develop their own plans for obstetric care to manage their medical records and ensure that the necessary information is shared with healthcare providers and shelter staff [21].

General Perinatal Medical Centers (GPMCs) are medical institutions in Japan designated by the Ministry of Health, Labour and Welfare that provide advanced medical care for mothers and newborns during the perinatal period. They are equipped with a maternity ward that includes a maternal–fetal intensive care unit (MFICU) and a neonatal ward that includes a neonatal intensive care unit co-located with the MFICU-equipped maternity ward. These centers maintain a 24/7 system for accepting maternal and neonatal transfers and can provide perinatal care for high-risk pregnancies and advanced neonatal medical care [22]. In Japan, the importance of treatment and care for high-risk pregnancies and high-risk newborns is increasing [23], and GPMCs play a central role in providing care [24].

The Maternal and Child Health Handbook (MCHH) was developed in Japan and is now used in approximately 50 countries, primarily in Asia and Africa [25]. The MCHH is a notebook that contains health information about mothers and infants from pregnancy through early childhood, including records of prenatal and infant health checkups. The MCHH is an important self-help measure for pregnant women in Japan and could help them to prepare for disaster-related disruptions to communication infrastructure and breakdown in information sharing. The MCHH can be used to share personal information, facilitating smooth collaboration with medical and support organizations and ultimately safeguarding the health of both the pregnant woman and the fetus. Reports on MCHH use following the Great East Japan Earthquake indicate its effectiveness in post-disaster maternal and child health activities [26]. Consolidating and providing easy access to a woman’s pregnancy history can help to ensure continuous and consistent care [27]. Various positive effects of MCHH use on maternal and child health management from the prenatal to the postnatal period have been demonstrated. For example, the MCHH was introduced in Indonesia in 1993 with the cooperation of the Japan International Cooperation Agency to address high maternal and infant mortality rates [28]. Studies indicate that mothers using the MCHH tend to have higher levels of knowledge about pregnancy and infant feeding. Furthermore, mothers who use the MCHH show higher rates of maternal and child health service usage, suggesting an effect on behavior as well as knowledge [29]. In Vietnam, interventions using the MCHH have demonstrated a substantial increase in the proportion of pregnant women receiving the recommended three or more antenatal care visits and in the rate of exclusive breastfeeding for the first 6 months postpartum [30]. In Japan, disaster-related surveys have reported that pregnant women who use the free description section of the MCHH tend to have greater knowledge regarding the disaster emergency message dial, mobile phone emergency message board, and hazard maps [31]. In recent years, recognition of the MCHH’s utility in understanding maternal and child health status, but also its potential for loss during disasters, have led to efforts to promote cloud-based and digital versions [32]. However, considering the possibility of large-scale power outages and damage to communication infrastructures during disasters, the use of the paper-based notebook version of the MCHH to manage information remains essential.

The objective of this pilot study was to clarify the current implementation status of disaster prevention education (DPE) provided to pregnant women by medical institutions, as well as the implementation status and related factors concerning education about MCHH use during disasters. Although the implementation rate of DPE in medical facilities for pregnant and postpartum women increased to 44.4% following the Great East Japan Earthquake, it remains below 50% [33]. A survey of obstetric facilities in the Chugoku and Shikoku regions of Japan found that only 20% of facilities provided disaster preparedness education to pregnant women [34]. Furthermore, a literature review on disaster preparedness during pregnancy indicated that although healthcare providers recognized the importance of disaster-related guidance in antenatal care, few implemented it. The reasons cited for this lack of information provision included insufficient disaster knowledge among nursing staff and difficulties securing the necessary personnel, time, and educational space [35]. Despite some researchers emphasizing the need for healthcare professionals to instruct pregnant women to carry their MCHH at all times, not just during checkups or hospital visits [36], less than 25% of facilities implement this instruction [37].

Considering the risks of adverse pregnancy outcomes owing to disasters, accessible and effective DPE during pregnancy is extremely important. Despite this need, DPE for pregnant and postpartum women remains in the developmental stage [38,39,40,41]. In particular, few studies have specifically focused on the operational status of the MCHH [26,31], a unique tool in maternal health, during disasters. Clarifying how medical institutions use this handbook for disaster preparedness is a novel aspect of this study, and could offer a model for other countries that use similar patient-held records.

The aim of this pilot study was to investigate the current state of DPE using the MCHH in medical institutions and the challenges faced by healthcare professionals. The findings may help to elucidate factors such as the extent to which medical institutions provide DPE to pregnant women and the content of that education, as well as the challenges involved. Furthermore, they may provide insights into the educational content needed to empower pregnant women to prepare for disasters and use their MCHH effectively, and provide preliminary findings for exploring related factors.

## 2. Materials and Methods

### 2.1. Data Collection

This study was conducted in three prefectures in Japan, selected using purposive sampling based on their distinct disaster histories and geography. The selected areas comprised (1) a prefecture in which severe damage from a Nankai Trough earthquake (Pacific coast) was predicted; (2) a prefecture that had had severe damage from a major inland earthquake in 2016; and (3) a prefecture located on the Sea of Japan coast that has not had any large-scale disasters in the last 5 years. Within these three selected prefectures, we targeted all 101 medical facilities participating in the Japan Obstetric Compensation System for Cerebral Palsy (JOCSCP). Specific prefecture names are withheld to protect the anonymity of the participating facilities.

An anonymous, self-administered questionnaire survey was conducted of the managers responsible for maternal health education departments at these 101 facilities. The JOCSCP is a system established to provide prompt financial compensation to children (and their families) who develop severe cerebral palsy related to childbirth. The aim of the JOCSCP is to assist the early resolution of disputes and the improvement of the quality of obstetric medical care through root cause analysis of cases and the proposal of recurrence prevention measures [42]. An original questionnaire was developed based on previous studies [43,44,45]. The questionnaire development process included a comprehensive literature review; selection of potential survey items; discussions among researchers regarding item relevance, clarity, and consistency with the research purpose; and content improvement following a review by two faculty members specializing in maternal–perinatal nursing to ensure face and content validity. These experts verified that the items accurately reflected the current disaster prevention guidelines and clinical reality in Japan.

The questionnaire consisted of three main sections. First, regarding facility classification, respondents were asked to indicate whether their facility was a designated disaster base hospital (0: No, 1: Yes) and their facility type (GPMC or regional perinatal medical center [RPMC]; general hospital not designated as a GPMC or RPMC; a clinic; or a midwifery center). Second, regarding DPE for pregnant women, the survey included 29 items about DPE provision. These items covered areas such as the patient’s need to be able to explain the results of previous antenatal checkups and their current physical condition, as well as the need to know the location of neighboring obstetric facilities. All 29 items are shown in Table 1. A complete list of these items is also provided in Supplementary Material. These items were selected to represent the essential functions of the MCHH during disasters. Third, respondents at facilities that had not implemented DPE were asked about their future implementation plans (1: considering implementing DPE, 2: see the need but have no plans to implement it, 3: no plans to implement it). Those who selected responses 2 or 3 were further asked to identify barriers to implementation (e.g., insufficient time, insufficient personnel, unclear what information to convey, or no available media). Data collection was conducted from August to November 2020. This period coincided with the COVID-19 pandemic, providing a unique historical context regarding the challenges to medical institutions during a global health crisis.

### 2.2. Data Analysis

We stratified the responses of the 28 respondents by those who had not yet implemented DPE and those who had. To examine factors related to the implementation of education explaining that the MCHH can be used to track the pregnancy progress and serve as a substitute for identification, and that it should be carried at all times (i.e., guidance on MCHH usage during disasters (focusing on pregnancy progress, identification, and carrying the handbook)), we categorized respondents into two categories based on whether or not DPE on MCHH was conducted. Specifically, responses were reclassified as a binary variable, with a coding of “No” assigned to facilities that either reported no DPE implementation at the facility level or implemented DPE but did not cover specific questionnaire items. A coding of “Yes” was assigned to facilities that confirmed DPE implementation. There were five options for the duration of the program: “within 15 min,” “15 to 30 min,” “30 min to 1 h,” “1 to 2 h,” and “more than 2 h.” However, as no facility selected the response “more than 30 min,” two categories were used: “within 15 min” and “15 to 30 min.”

Descriptive statistics were calculated to analyze DPE implementation. Specifically, the distribution of data was summarized by calculating the percentage of each survey item that was composed of each response category. Next, “guidance on MCHH usage during disasters (focusing on progress, identification, and carrying)” was set as the objective variable and analyzed using Fisher’s direct probability test to examine its association with the other variables. All data entry, tabulations, and statistical analyses were performed using IBM SPSS Ver. 29 (Armonk, NY, USA). The significance level was 5%. Given the exploratory nature of this pilot study, no statistical adjustment for multiple comparisons (e.g., Bonferroni correction) was performed. Consequently, the results should be interpreted as hypothesis-generating rather than confirmatory.

### 2.3. Ethical Considerations

This study was conducted following the 1995 Declaration of Helsinki (revised in Seoul in 2008) and with the consent of the Kobe City University of Nursing Research Ethics Committee (No: 2019-2-17-01). Participants were given a written explanation of the study, including the respect for the participants’ freedom to refuse or withdraw participation, anonymity, protection of their privacy, and other aspects of the study. An informed consent document explaining the research purpose and methods, and the fact that all responses would be anonymous, was mailed to participants. The return of the completed questionnaire was considered to imply consent for participation. To ensure participants’ anonymity, no identifying information was intentionally collected.

## 3. Results

### 3.1. Overview of Participating Facilities

Of the 101 facilities contacted, responses were obtained from administrators of departments responsible for maternal health education at 28 facilities (27.7%), and all 28 responses were included in the analysis. Of these facilities, 12 (42.9%) were designated disaster base hospitals. Regarding facility type, 6 (21.4%) were GPMCs or RPMCs, 7 (25.0%) were general hospitals, 14 (50.0%) were clinics, and 1 (3.6%) was a midwifery center (Table 2).

### 3.2. Status of DPE Implementation

Among the 28 participating facilities, 9 (32.1%) had implemented DPE. Most of these facilities (66.7%) reported an implementation time of less than 15 min, and none exceeded 30 min (Table 2).

### 3.3. Future Plans for Facilities Not Conducting DPE (Multiple Responses)

Among the 19 facilities not currently implementing DPE, 13 (68.4%) recognized the need for DPE but were unable to implement it, while 4 (21.1%) had no future plans for implementation. Common barriers cited by these facilities included uncertainty regarding appropriate educational content (*n* = 11) and staff shortages (*n* = 10).

### 3.4. Implementation Status and Related Factors of Guidance on MCHH Usage During Disasters

Fisher’s exact test was conducted to examine the relationship between the implementation of education on how to use the MCHH in a disaster (covering pregnancy progress, identification, and carrying) and other educational items. The results showed notable trends in the data distribution between several variables. Specifically, some DPE items were implemented exclusively by facilities that also provided MCHH guidance.

Regarding facility characteristics, guidance on MCHH usage during disasters showed a data distribution trend toward GPMCs and RPMCs (*n* = 5, 83.3%; *p* = 0.007). However, the 2 × 2 tables for other variables indicated the presence of zero cells.

Although a distribution difference was observed between the implementation of guidance on MCHH usage during disasters and specific educational contents, these variables also exhibited zero cells: the need to be able to explain the results of previous antenatal checkups and the patient’s current physical condition (*n* = 7, 100%; *p* < 0.001), and the need to know the location of several obstetrics and gynecology hospitals, clinics, and midwifery centers in the neighborhood, other than the hospital where the patient was being treated (*n* = 3, 100%; *p* = 0.026). Detailed results for all 29 items are shown in Table 1.

## 4. Discussion

The results of this exploratory pilot study provide preliminary insights into a potential relationship between the implementation of guidance on MCHH usage in DPE provided to pregnant women during disasters and the facility type (GPMC/RPMC vs. other facilities). Furthermore, despite the presence of zero cells in the contingency table owing to the small sample size, associations were also suggested for several variables for which the need for preparedness and response during disasters is broadly advocated, even in non-medical settings.

The observed tendency for PMC facilities to provide education on MCHH carrying and use may highlight the perceived importance of MCHH possession during disasters, despite the multifaceted and busy nature of these institutions. In particular, GPMCs, which are involved daily in diverse operations, and frequently provide emergency responses and interventions for high-risk pregnant women and fetuses, may have a heightened awareness of the essential role of information sharing during disasters, potentially contributing to the observed results. Similar challenges regarding the disruption of the continuum of care and loss of medical information have been reported in other countries in disaster contexts such as hurricanes and wildfires [13,14], as well as earthquakes [11,16,17]. In fact, previous studies have reported that during the Great East Japan Earthquake, the MCHH served as a vital backup for medical records when electronic systems were rendered inaccessible owing to power outages and tsunami damage, thereby ensuring continuity of care [10]. This historical lesson has likely increased the motivation of PMCs to instruct pregnant women to carry their handbooks. Although the presence of zero cells in the contingency table necessitates very cautious interpretation of the results, the statistical trends suggested a link between the implementation of education on MCHH use during disasters and several other variables. However, these associated variables mainly comprised general education content for pregnant women, such as the need to explain their pregnancy history and the importance of knowing the location of appropriate medical institutions other than their primary institution, or general disaster preparedness information, such as clarifying family communication methods during disasters and confirming evacuation sites in their residential area. Furthermore, it has been widely recognized that natural disasters and public health crises substantially affect the health of pregnant women; however, research and educational initiatives specifically targeting emergency preparedness for this population remain limited, indicating a notable discrepancy between theoretical guidelines and actual implementation at the medical front [46,47]. This global challenge aligns with the current situation in Japan. For instance, studies in Indonesia have also highlighted the necessity of improved disaster management education for mothers, as baseline preparedness often remains insufficient [40,41]. Indeed, a 2022 survey of perinatal medical facilities in Japan reported that the implementation rate of DPE for pregnant and postpartum women was approximately 33%, and the content mainly focused on the routine carrying of items such as the MCHH, confirmation of evacuation routes and communication methods, and promotion of breastfeeding [37]. Considering these points, we suggest that among the medical institutions that conduct DPE included in this study, general disaster preparedness awareness may be promoted alongside specific education on MCHH use during disasters.

## 5. Limitations

This study had several limitations. First, the most important limitation is the low response rate (27.7%) and the resulting small sample size (*N* = 28), which raises serious concerns regarding selection bias and representativeness. It is highly plausible that facilities with a pre-existing interest in disaster education were more likely to respond (non-response bias), potentially overestimating the actual implementation status across general facilities. The survey coincided with the COVID-19 pandemic (August to November 2020), during which many medical institutions were overwhelmed with the need to provide infection control measures, which likely contributed to the low response rate. Second, a zero cell was observed in one of the 2 × 2 contingency table cells in Fisher’s exact test examining the association between the presence of education on MCHH use during disasters and other variables. This means that the results must be interpreted with considerable caution and assumed to indicate preliminary trends rather than definitive evidence, because the presence of a zero cell can affect the evaluation of the association between variables. Third, owing to the sample size limitations, we were limited to univariate analysis, and were unable to control for the effects of potential confounding factors using multivariate analysis. Furthermore, regarding the questionnaire used in this study, although content validity was confirmed through expert review, formal psychometric testing (e.g., factor analysis, internal consistency reliability) was not conducted. This is a limitation inherent to a self-developed checklist designed to assess factual implementation status rather than latent constructs. Similarly, stratified analysis could not be performed. Therefore, these results may be subject to bias from unmeasured or uncontrolled confounding factors. In particular, important potential confounders such as respondents’ age and years of professional experience, which may have influenced the observed associations, were not investigated. Consequently, there are important limitations to the generalizability of these findings, which are strictly exploratory and preliminary. Given the results, future research should revise the survey content and use a substantially larger sample size for validation.

## 6. Conclusions

Despite the above-mentioned limitations, this study highlights a critical gap in disaster preparedness even among advanced perinatal centers, and thus the findings have important clinical implications. Despite the limitations of this small-scale pilot study, the findings suggest that GPMCs and RPMCs may be more likely to provide education on MCHH use (including carrying) in disasters and may also be more proactive in promoting general disaster preparedness measures beyond MCHH usage. However, considering the low implementation rate of such education even among the responding facilities, there is a need for more practical and widespread dissemination of better DPE measures that include MCHH use. Additional studies with larger, representative samples are needed to confirm these preliminary findings. Targeted DPE could improve self-help in pregnant women and increase their opportunities for mutual support. This could help to reduce negative effects on health and the risk of adverse pregnancy outcomes during disasters.

## Figures and Tables

**Table 1:** Relationship between DPE content and MCHH usage education in disasters (*N* = 28).

				Guidance on MCHH Usage During Disasters				
Items	Category	Total		Not Implemented (*n* = 19)		Implemented (*n* = 9)		
		*n*	%	*n*	%	*n*	%	*p*-Value
*Visiting obstetric medical facilities during disasters*								
Covering issues such as the need to be able to explain the results of previous antenatal checkups and patient’s current physical condition	No	21	75.0	19	90.5	2	9.5	<0.001
	Yes	7	25.0	0	0.0	7	100.0	
The need to know the location of several obstetrics and gynecology hospitals, clinics, and midwifery centers in the neighborhood, other than the hospital where the patient is being treated.	No	25	89.3	19	76.0	6	24.0	0.026
	Yes	3	10.7	0	0.0	3	100.0	
*Regarding contact methods in the event of a disaster*								
The need for clear communication methods in the event of a disaster	No	21	75.0	19	90.5	2	9.5	<0.001
	Yes	7	25.0	0	0.0	7	100.0	
The need for families to decide how to keep in touch with each other in the event of a disaster	No	24	85.7	19	79.2	5	20.8	0.006
	Yes	4	14.3	0	0.0	4	100.0	
Awareness of the disaster message dial	No	26	92.9	19	73.1	7	26.9	0.095
	Yes	2	7.1	0	0.0	2	100.0	
Awareness of the mobile phone emergency message board	No	27	96.4	19	70.4	8	29.6	0.321
	Yes	1	3.6	0	0.0	1	100.0	
The need to confirm how to communicate with daycare centers, kindergartens, and schools	No	26	92.9	19	73.1	7	26.9	0.095
	Yes	2	7.1	0	0.0	2	100.0	
The need to confirm the location of pay phones and have coins ready	No	27	96.4	19	70.4	8	29.6	0.321
	Yes	1	3.6	0	0.0	1	100.0	
*Information regarding evacuation shelters and evacuation routes from your home or workplace in the event of a disaster*								
The need to confirm evacuation centers in the area of residence	No	20	71.4	19	95.0	1	5.0	<0.001
	Yes	8	28.6	0	0.0	8	100.0	
The need to check routes to evacuation sites	No	25	89.3	19	76.0	6	24.0	0.026
	Yes	3	10.7	0	0.0	3	100.0	
The need to check hazard maps	No	24	85.7	19	79.2	5	20.8	0.006
	Yes	4	14.3	0	0.0	4	100.0	
*Disaster preparedness measures within the home*								
The need to check the earthquake resistance of the house	No	26	92.9	19	73.1	7	26.9	0.095
	Yes	2	7.1	0	0.0	2	100.0	
The need to ensure that furniture items do not fall over during an earthquake	No	24	85.7	19	79.2	5	20.8	0.006
	Yes	4	14.3	0	0.0	4	100.0	
The need to take measures to prevent falling objects	No	24	85.7	19	79.2	5	20.8	0.006
	Yes	4	14.3	0	0.0	4	100.0	
The need for measures to prevent glass from shattering	No	26	92.9	19	73.1	7	26.9	0.095
	Yes	2	7.1	0	0.0	2	100.0	
The need to sleep in a safe place that will not be under falling objects during an earthquake	No	25	89.3	19	76.0	6	24.0	0.026
	Yes	3	10.7	0	0.0	3	100.0	
The need to identify the safest place in the house	No	26	92.9	19	73.1	7	26.9	0.095
	Yes	2	7.1	0	0.0	2	100.0	
Preparation of necessary supplies for pregnant women (including general disaster preparedness goods, sanitary napkins, etc.)	No	20	71.4	19	95.0	1	5.0	<0.001
	Yes	8	28.6	0	0.0	8	100.0	
*Using the Maternal and Child Health Handbook*								
To enter the test results in the Maternal and Child Health Handbook	No	21	75.0	19	90.5	2	9.5	<0.001
	Yes	7	25.0	0	0.0	7	100.0	
To explain which part of the Maternal and Child Health Handbook can be filled in by the mother	No	22	78.6	19	86.4	3	13.6	<0.001
	Yes	6	21.4	0	0.0	6	100.0	
*Nutritional intake of children during disasters*								
Breastfeeding mothers are encouraged to continue breastfeeding after the disaster	No	19	67.9	19	100.0	0	0.0	<0.001
	Yes	9	32.1	0	0.0	9	100.0	
Recommends breastfeeding daily because breast milk is the right temperature, clean, etc., in times of disaster	No	22	78.6	19	86.4	3	13.6	<0.001
	Yes	6	21.4	0	0.0	6	100.0	
Recommends stockpiling powdered or liquid infant formula	No	26	92.9	19	73.1	7	26.9	0.095
	Yes	2	7.1	0	0.0	2	100.0	
*How to respond if you experience signs of labor or physical changes during a disaster*								
If there is little fetal movement, etc., see a doctor immediately	No	22	78.6	19	86.4	3	13.6	<0.001
	Yes	6	21.4	0	0.0	6	100.0	
The condition of the fetus should be indicated by fetal movements	No	23	82.1	19	82.6	4	17.4	<0.001
	Yes	5	17.9	0	0.0	5	100.0	
If the belly is distended, the first thing to do is to lie down	No	24	85.7	19	79.2	5	20.8	0.006
	Yes	4	14.3	0	0.0	4	100.0	
If your water breaks, lie down with a clean napkin, etc.	No	26	92.9	19	73.1	7	26.9	0.095
	Yes	2	7.1	0	0.0	2	100.0	
*Experiences of pregnant women affected by the disaster*								
Provide opportunities to learn directly from pregnant women who have experienced disasters	No	28	100.0	19	67.9	9	32.1	
	Yes	0	0.0	0		0		
Provide pamphlets and brochures with stories about their experiences	No	27	96.4	19	70.4	8	29.6	0.321
	Yes	1	3.6	0	0.0	1	100.0	

Fisher’s exact test was used for analysis. DPE, disaster prevention education; MCHH, Maternal and Child Health Handbook.

**Table 2:** Relationship between facility type, DPE implementation time, and MCHH education in disasters (*N* = 28).

				Guidance on MCHH Usage During Disasters				
Items	Category	Total		Not Implemented (*n* = 19)		Implemented (*n* = 9)		
		*n*	%	*n*	%	*n*	%	*p*-Value
Disaster base hospital	No	16	57.1	12	75.0	4	25.0	0.432
	Yes	12	42.9	7	58.3	5	41.7	
General perinatal medical center/Regional perinatal medical center	No	22	78.6	18	81.8	4	18.2	0.007
	Yes	6	21.4	1	16.7	5	83.3	
Hospital	No	21	75.0	13	61.9	8	38.1	0.371
	Yes	7	25.0	6	85.7	1	14.3	
Clinic	No	14	50.0	7	50.0	7	50.0	0.103
	Yes	14	50.0	12	85.7	2	14.3	
DPE implementation time: <15 min	No	22	78.6	16	72.7	6	27.3	0.352
	Yes	6	21.4	3	50.0	3	50.0	
DPE implementation time: 15–30 min	No	25	89.3	17	68.0	8	32.0	1.000
	Yes	3	10.7	2	66.7	1	33.3	

Fisher’s exact test was used for analysis. DPE, disaster prevention education; MCHH, Maternal and Child Health Handbook.

## Data Availability

The data presented in this study are not available due to ethical and legal restrictions related to the protection of personal information.

## Supplementary Materials

The following supporting information can be downloaded at: https://www.mdpi.com/article/10.3390/nursrep16020071/s1, Supplementary File S1: Survey Items.

## Public Involvement Statement

There was no public involvement in any aspect of this research.

## Guidelines and Standards Statement

Reporting Summary: This manuscript was drafted against the Strengthening the Reporting of Observational Studies in Epidemiology (STROBE) guidelines for cross-sectional research [48].

## Abbreviations

The following abbreviations are used in this manuscript:

DPE	Disaster prevention education
MCHH	Maternal and Child Health Handbook
GPMC	General perinatal medical center
RPMC	Regional perinatal medical center
MFICU	Maternal–fetal intensive care unit
JOCSCP	Japan Obstetric Compensation System for Cerebral Palsy
EM-DAT	Emergency Events Database
