# Supplementary material for: Implementation of Disaster Prevention Education and Maternal and Child Health Handbook Guidance for Pregnant Women in Japanese Medical Institutions: A Pilot Study
Source: Nurs Rep. 2026 Feb 18;16(2):71. doi: 10.3390/nursrep16020071 (PMC12943356; doi:10.3390/nursrep16020071)
Supplement: Supplementary file 1 [file nursrep-16-00071-s001.zip › nursrep-4090465-supplementary.pdf]

Supplementary S1: Survey Items

1. **Facility classification**

   Disaster base hospital (0: No, 1: Yes), facility classification (general perinatal medical center [GPMC] or regional perinatal medical center [RPMC], general hospital not designated as a GPMC or RPMC, clinic, midwifery center)

2. **Disaster prevention education for pregnant women**

   (1) Visiting obstetric medical facilities during disasters (0: No, 1: Yes)

   Covering issues such as the need to be able to explain the results of previous antenatal checkups and the patient's current physical condition

   The need to know the location of several obstetrics and gynecology hospitals, clinics, and midwifery centers in the neighborhood, other than the hospital where the patient is being treated

   (2) Regarding contact methods in the event of a disaster (0: No, 1: Yes)

   The need for clear communication methods in the event of a disaster

   The need for families to decide how to keep in touch with each other in the event of a disaster

   Awareness of the disaster message dial (171)

   Awareness of the mobile phone emergency message board

   The need to confirm how to communicate with daycare centers, kindergartens, and schools

   The need to confirm the location of pay phones and have coins ready

   (3) Information regarding evacuation shelters and evacuation routes from your home or workplace in the event of a disaster (0: No, 1: Yes)

   The need to confirm evacuation centers in the area of residence

   The need to check routes to evacuation sites

   The need to check hazard maps

   (4) Disaster preparedness measures within the home (0: No, 1: Yes)

   The need to check the earthquake resistance of the house

   The need to ensure that furniture items do not fall over during an earthquake

   The need to take measures to prevent falling objects

   The need for measures to prevent glass from shattering

The need to sleep in a safe place that will not be under falling objects during an earthquake

The need to identify the safest place in the house

Preparation of necessary supplies for pregnant women (including general disaster preparedness goods, sanitary napkins, etc.)

(5) Using the Maternal and Child Health Handbook (0: No, 1: Yes)

To enter the test results in the Maternal and Child Health Handbook

To explain which part of the Maternal and Child Health Handbook can be filled in by the mother

(6) Nutritional intake of children during disasters (0: No, 1: Yes)

Breastfeeding mothers are encouraged to continue breastfeeding after the disaster

Recommends breastfeeding daily because breast milk is the right temperature, clean, etc., in times of disaster

Recommends stockpiling powdered or liquid infant formula

(7) How to respond if you experience signs of labor or physical changes during a disaster (0: No, 1: Yes)

If there is little fetal movement, etc., see a doctor immediately

The condition of the fetus should be indicated by fetal movements

If the belly is distended (uterine contractions), the first thing to do is to lie down

If your water breaks, lie down with a clean napkin, etc.

(8) Experiences of pregnant women affected by the disaster (0: No, 1: Yes)

Provide opportunities to learn directly from pregnant women who have experienced disasters

Provide pamphlets and brochures with stories about their experiences
